# Prevalence of Idiopathic Cyclic Edema in Women with Lower Limb Lymphedema

**DOI:** 10.3390/jcm7010002

**Published:** 2017-12-25

**Authors:** Jose Maria Pereira de Godoy, Henrique Jose Pereira de Godoy, Lívia Maria Pereira de Godoy, Maria de Fatima Guerreiro Godoy

**Affiliations:** 1 Department of Cardiology and Cardiovascular Surgery, Medicine School in São José do Rio Preto (FAMERP), CNPq (National Council for Research and Development), São José do Rio Preto 15025120, Brazil; 2 Medicine School of Universidade Federal do Mato Grosso-Cuiabá-UFMT and Research Group in the Clínica Godoy, São José do Rio Preto 15025120, Brazil; henriquegodoy95@gmail.com; 3 Clinic General in Medicine School of Marilia-São Paulo-Brazil (FANEMA) and Researcher Group of the Clínica Godoy, São José do Rio Preto 15025120, Brazil; godoylmp@gmail.com; 4 Occupational Therapist Professor of the Post-Graduate Strictu Sensu in Medicine School in São José do Rio Preto (FAMERP) and Research Group in the Clínica Godoy, São José do Rio Preto 15025120, Brazil; mfggodoy@gmail.com

**Keywords:** idiopathic cyclic edema, lymphedema, edema, treatment

## Abstract

Cyclic edema is a clinical condition in women that leads to fluid retention in the orthostatic position. The aim of the present study was to evaluate the prevalence of idiopathic cyclic edema in women with lower limb lymphedema. The prevalence of idiopathic cyclic edema was evaluated in a retrospective study of 100 consecutive female patients submitted to leg lymphedema treatment at the Clínica Godoy. The diagnosis of lymphedema was clinical, based on patient history and a physical examination. Patients with clinical stage II lymphedema were included in the study with those in stages I and III being excluded. The diagnosis of idiopathic cyclic edema was based on the patient’s history and fluid retention of more than one kilogram between 7:00 a.m. and 5:00 p.m. Clinical signs of this disease include difficulty removing rings in the morning that becomes easier during the course of the day, waking up with a swollen face, and abdominal discomfort during the day. After diagnosing cyclic edema, a therapeutic test was performed using aminaphtone or calcium dobesilate with which fluid retention was reduced to less than 300 g during the same period. The patients were instructed to drink liquids only when they were thirsty.

## 1. Introduction

Lymphedema is a clinical condition that results from the accumulation of macromolecules in the interstitial space, leading to fluid retention. Primary (congenital) or acquired disorders of the lymphatic system may lead to the development of lymphedema [1]. The diagnosis of lymphedema, usually based on clinical history and physical examination, is supported when there is doubt with complementary examinations such as lymphoscintigraphy, the gold standard exam in the evaluation of anatomical and functional alterations in lymphatic vessels. Exams such as a duplex ultrasound to exclude other causes of associated edema may be necessary to rule out specific venous disorders [1,2].

Primary or secondary lymphedemas may be aggravated by other disorders such as erysipelas and radiotherapy that can lead to further changes in lymphatic vessels [3]. Other conditions such as heart failure, hypoproteinemia, renal failure, and hepatic failure interfere with the dynamics of interstitial flow [4]. However, poorly assessed conditions such as idiopathic cyclic edema may interfere with lymph production and lead to a hyperdynamic status and consequently fluid retention and accumulation [4,5,6].

Cyclic edema is a clinical condition in women that leads to fluid retention in the orthostatic position [4]. Diagnosis is generally clinical, based on a history of body edema associated with the effects of gravitational pressure [6]. In the morning, carriers often complain about edema of the hands and face and have difficulty removing rings from their fingers; during the day, it becomes easier to take their rings off. The patients are requested to weigh themselves in the morning and in the evening with their bladder and stomach empty in order to accurately evaluate weight gain during the day; an accumulation of as much as 800 g in one day is reported by some patients. During the day, they are advised to drink about two liters of liquid, since about 50–60% of the volume of fluids consumed is usually retained in the body [6]. Another possible evaluation is the Landis test, but this is not a routine test [4,6].

As far as we know, studies evaluating the effects of cyclic edema in patients with lymphedema have not been described [5]. The aim of the present study was to evaluate the prevalence of idiopathic cyclic edema in women with lower limb lymphedema.

## 2. Materials and Methods

A retrospective study was performed to identify the prevalence of idiopathic cyclic edema in 100 consecutive female patients submitted to leg lymphedema treatment at the Clínica Godoy (105890/2017). The diagnosis of lymphedema was clinical, based on patient history and a physical examination. It is done routinely with tests that measure volume such as volumetry by water displacement, bioimpedance, perimetry, and body weight. Patients with clinical stage II lymphedema were included in the study with those in stages I and III being excluded. The diagnosis of idiopathic cyclic edema was based on the patient’s history and an assessment of fluid retention characterized by an increase in weight of more than one kilogram between 7:00 a.m. and 5:00 p.m. Clinical signs included difficulty removing rings in the morning that became easier during the course of the day, waking up with a swollen face, and abdominal discomfort during the day. After the diagnosis of cyclic edema, a therapeutic test was performed; by prescribing aminaphtone or calcium dobesilate, fluid retention was reduced to less than 300 g during the same period. The dosage of aminaphtone was 75 mg three times daily and dobesilate calcium 500 mg twice daily. The result was visible within two to three days and the treatment was maintained for about 6 months. The patients were instructed to drink liquids only when they were thirsty.

## 3. Results

The age of these patients ranged from 21 years to 76 years with an average of 40.2 years. It was found that 9% of the participants had idiopathic cyclic edema. A therapeutic response with a reduction in edema occurred within two days. Four patients took aminaphtone and five patients took calcium dobesilate as monotherapy. As monotherapy, no response was obtained in one patient treated with aminaphtone and in one patient treated with calcium dobesilate; however, when the two drugs were combined, both patients responded with reductions in weight.

## 4. Discussion

Lymphedema is a chronic clinical condition and may have other aggravating factors, such as idiopathic cyclic edema, as described in this study. Therefore, in addition to the primary or secondary alterations of the lymphatics seen in lymphedema, other causes that may contribute to further impairment should be evaluated, including venous insufficiency, hypoproteinemia, cardiac insufficiency, renal insufficiency, and hepatic insufficiency [4]. However, in addition to these, there are other clinical conditions that are relatively unknown or underdiagnosed and are important in the approach for these patients. Cyclic edema is an underdiagnosed condition that was detected in 9% of the women in this study [4,5,6].

The prevalence of idiopathic cyclic edema associated with lower limb lymphedema has not been reported in the literature nor has any specific therapeutic approach to these patients been reported. A study using lymphoscintigraphy shows that there is a pattern of hyperflow related to cyclic edema in the lymphatic system, and thus the already deficient system can become overloaded [5].

There are a few reports that describe a therapeutic approach to patients with idiopathic cyclic edema. One of them evaluated the response of one patient with lipolymphedema and idiopathic cyclic edema to calcium dobesilate. This patient was treated with medication only. This report shows the importance when treating lymphedema to control other types of edema that may be the cause of the swelling [7].

Idiopathic cyclic edema is a therapeutic challenge when treating lymphedema because we do not entirely understand its pathophysiology. Today there are drugs that help control edema. There is probably a mechanical cause associated with a functional condition related to changes in the permeability of the vessels. Failure to control cyclic edema leads to a hyperdynamic pattern of lymph production that may impede treatment of lymphedema.

Another aspect of idiopathic cyclic edema is that it is poorly described in the literature. No cases of idiopathic cyclic edema have been found in men. Some drugs such as calcium dobesilate, aminaphtone, and ginkgo biloba have been shown to control 50–70% of the edema during the daytime and should be used for six months [6,7]. Relapse is very high when these drugs are used for a short period of time of around one month, making it difficult to control and maintain the results of lymphedema treatment. These patients should be counseled about a possible relapse of the edema, a condition that is often perceived by them, and recommended to take the medication for another period. There is no study in the literature defining the time of treatment or the relapse rate. A fundamental detail is that these patients should not drink excessive liquid as this hinders control.

In summary, besides the anatomical alterations of the lymphatic system, functional alterations that can lead to an overload of this system must be evaluated and treated simultaneously. Failure to do so may make lymphedema treatment difficult or even unfeasible.

## 5. Conclusions

Women with primary or secondary lymphedema should be evaluated for the possibility of having idiopathic cyclic edema. Moreover, hyperdynamic-type lymphedema caused by this form of edema should be considered.
